# Parental Divorce Process and Post-Divorce Parental Behaviors and Strategies: Examining Emerging Adult Children’s Attachment-Related Anxiety and Avoidance

**DOI:** 10.3390/ijerph191610383

**Published:** 2022-08-20

**Authors:** Klara Smith-Etxeberria, Irune Corres-Medrano, Itziar Fernandez-Villanueva

**Affiliations:** Department of Basic Psychological Processes and Development, Faculty of Psychology, University of the Basque Country, 20018 San Sebastián, Spain

**Keywords:** parental divorce, coparental cooperation and respect, post-divorce interparental conflict, parental emotional state and instability, positive parenting, attachment-related anxiety and avoidance, emerging adult children

## Abstract

The main goal of this study was to examine the role of parental behaviors during both the process of divorce and the post-divorce period on emerging adult children’s attachment-related anxiety and avoidance. Specifically, we analyzed how recalled coparental respect and cooperation, interparental conflict, positive parenting strategies, and both parents’ emotional state and instability from adult children’s perspective during the divorce process and the post-divorce period were associated with emerging adult children’s current attachment representations. Our sample consisted of 173 emerging adults (Mage = 22.01). The results of this study demonstrate that paternal coparental respect and cooperation along with freedom provided by the mother to talk about the father during the divorce process and post-divorce period were both related to lower attachment-related avoidance. Our findings also confirm a significant link between some paternal positive attitudes during the divorce process (i.e., freedom provided by the father to talk about the mother) and low attachment-related anxiety. Overall, the results of this research confirm that beyond divorce perse, several variables surrounding the divorce process better explain variations in adult children’s attachment representations, which contribute to better comprehending the effects of parental divorce.

## 1. Introduction

Every year, a growing number of children undergo their parents’ divorce. For example, in Spain, in approximately 60% of all divorce cases, divorcing couples have both underage and overage children [1]. Empirical research has shown that parental divorce brings several negative consequences in both the short and the long term. Compared to children from non-divorced families, both children and adolescents show more internalizing and externalizing problems, lower academic achievement, or greater attachment insecurity in the short-term [2,3,4,5]. In the long term, there is also ample evidence about parental divorce as a risk factor for adult children. Moreover, on average, adult children of divorce show lower well-being levels, higher divorce rates, poor marital quality, less positive parent–child relationships, and more insecure attachment styles and more negative internal working models about intimate relationships than adult children from non-divorced families [6,7,8,9,10].

Early attachment interactions and caregiving experiences guide social and close relationships throughout the life span, and shape the development of working models about intimate relationships in adulthood [11,12]. Nonetheless, the parental marital relationship quality and dissolution may also contribute to adult children’s attachment representations and the quality of their subsequent relationships [13,14]. Since interparental relationships are frequently the first model of romantic relationships for children, experiencing the dissolution of the parental couple relationship could harm young adult children’s internal working models and security in their intimate relationships [14]. Furthermore, relationship disruptions in the family of origin during childhood might influence the ways in which young adults approach romantic relationships and their mental representations regarding these relationships [14]. That is, parental divorce or separation might lead to the development of maladaptive schemas about intimate relationships, such as negative expectations towards being abandoned by others, which in turn has been positively associated with anxious and avoidant attachment styles in adult children [15].

A large number of studies have investigated the impact of parental divorce on adult children’s attachment, theoretically because of changes in the parent–child attachment relationship following divorce [16,17]. That is, stressors that accompany parental divorce might affect parents’ ability to be sensitive to their children’s needs, leading to less secure parent–child attachment bonds. These negative changes in parent–child attachment relationships might in turn result in a more insecure romantic attachment among adult children [16,17]. 

In this vein, although several studies have consistently concluded that adult children from divorced families, and especially women, are more likely to be insecurely attached than their counterparts from non-divorced families [9,13,18,19], the results are mixed. Furthermore, some other studies have failed to replicate these results, by not finding significant associations between parental divorce and adult children’s attachment-related strategies or representations [20,21,22,23]. These controversial results regarding the effects of parental divorce on adult children’s attachment-related representations might be because most of these studies have analyzed parental divorce as a single dichotomous variable by uniquely asking participants whether their parents were separated or divorced [22]. These prior studies have failed to examine circumstances surrounding the divorce process and the post-divorce period that might better explain variations in the effects of parental divorce on adult children’s attachment-related representations. Thus, the lack of conclusive findings may indicate that there is ample variability in the nature of parental divorce experiences, which might have a global effect on adult children’s attachment style, but that has not been identified [24]. Hence, one of our main aims is to shed further light on this issue, by identifying factors surrounding the divorce experience that might affect adult children’s attachment representations. 

According to the divorce stress-adjustment perspective [25], divorce in itself does not lead to negative consequences on family members, but rather to family life changes and stressful circumstances surrounding divorce that increase the risk of a variety of problems among children. These stressful factors refer to pre- and post-divorce interparental conflict levels, poorer relationship quality with the custodial parent, lower frequency of contact with the non-custodial parent [26,27], lower economic resources, and other stressful events, such as changing residence [28]. Moreover, this perspective defines divorce as a process in which several factors may moderate children’s reactions to divorce, and stressors related to this experience might mediate the association between parental divorce and the negative outcomes found in children that can persist into adulthood. Therefore, since factors that accompany this family transition event might help better comprehend adult children’s post-divorce adjustment, it is essential to examine the potential family functioning variables in divorced families that shape adult children’s post-divorce affective well-being. 

One post-divorce family functioning variable that has received a great attention in the literature refers to coparenting. Coparenting is understood not only as the collaboration in childrearing of two parental figures who share the responsibilities for at least one child, but also as the active attempt to support each other’s parenting while maintaining healthy and flexible bonds [29]. This kind of positive coparental relationship, also named coparental respect and cooperation, promotes a positive relationship between the child and the other parent, by also providing the child with the freedom to talk about one parent in front of the other, and leads to positive consequences in children [29,30,31]. 

There is robust evidence about the effects of these family functioning dynamics on both children and adults, although little is known about the potential roles that parents’ gender play. That is, very few studies have focused on separately investigating the effects of mothers’ and fathers’ coparenting behaviors [29]. Likewise, even if a few studies have examined the consequences of parental behaviors in the post-divorce period on adult children’s level of attachment security, to our knowledge, none have analyzed such influence by distinguishing specific attachment-related representations or dimensions (i.e., attachment-related anxiety and avoidance). Hence, in this study, we focus on the analysis of the effects of each parent’s coparental cooperation and respect behaviors [29] on adult children’s attachment-related anxiety and avoidance. 

Despite coparental interactions might sometimes be positive, these interactions in divorced families might also be negative or antagonistic in nature (i.e., high levels of conflict or/and unhealthy bonds with the child). In fact, even though parental divorce may be perceived as a relief from an adverse family context (e.g., high levels of interparental conflict) that prompts positive consequences [32], high levels of post-divorce interparental conflict have consistently been identified as a risk post-divorce family experience that accounts for variability in children’s maladjustment [33]. Indeed, in 20–25% of divorce cases, parents display highly conflicted coparental behaviors [34], which have been characterized by poor communication, little cooperation, mistrust, and disagreements in decision making. In this regard, there is extensive literature confirming its negative effect on children’s adjustment (for a review, see [33]). Post-divorce interparental conflict might be expressed through direct interactions, such as verbal and physical disputes, or indirectly, such as badmouthing the other parent to the child. This situation might cause negative outcomes in children by making them feel caught between both parents [33,34,35,36]. For example, it is quite frequent to put children in middle of the conflict by badmouthing the other parent to the child or by sending messages to the other parent through the child [28,37]. 

These negative family interactions, which may imply parents using the child in their conflict, also named triangulation or focusing on the child as the source of their disagreements (i.e., scapegoat), might lead to loyalty conflicts in the child, where being loyal to one parent involves being disloyal to the other [38,39]. This puts pressure on the child to take sides in the conflict through the attempt of one parent to form alliances with the child against the other parent [40]. This phenomenon has been conceptualized in different ways, such as parental interference, in which parents utilize behaviors, strategies, or actions to hinder and damage the relationship of the child with the other parent [41]. In an extreme parental interference situation, the child might lose contact with and reject the other parent [42]. 

Prior studies demonstrate that children who are exposed to such negative parental attitudes during the divorce process and the post-divorce period show anger, guilt, hostility, impulse control problems, low self-confidence and self-esteem, anxiety, depression, difficulties in personal and social relationships, and diminished academic performance (for a review, see [41]). Although there is less evidence regarding the long-term implications of this phenomenon in adult children, a few studies conducted in this developmental period have found negative emotional, psychological, and affective consequences. These include high anxiety and depression; low self-esteem, self-sufficiency, life satisfaction, and quality; along with negative parent–child relationships and insecure attachment in intimate relationships (for a review, see [41]). 

In addition to heightened interparental conflict in the post-divorce period of some families, stressors associated with the divorce process might also negatively affect parents’ well-being and emotional state, which, in turn, may have a negative impact on children’s adjustment. In fact, divorced parents report higher levels of depression, anxiety, and unhappiness, which might, in turn, negatively affect children’s adjustment to divorce [43], since a decrease in parental adjustment may influence the ability of parents to parent effectively [44]. After divorce, children are especially likely to need emotional support, and in the face of socioemotional and economic distress, parents are less likely to provide it effectively [43]. Indeed, the transition period following divorce has been characterized as a chaotic and stressful period for most families. This stressful period usually disturbs the parent–child relationship and leads to disruptions in parenting behaviors. Furthermore, research shows that divorce generally leads to a deterioration of positive parenting strategies (e.g., responsiveness) and an increase in negative parenting strategies (e.g., harshness) in both custodial and non-custodial parents [43]. For example, custodial parents are more prone to be inconsistent and more punitive following divorce, whereas noncustodial parents tend to have a diminished level of control over their children [43].

Thus, in the study of the effects of these family experiences, it is necessary to take into consideration divorce not as a uniform experience, but as a process in which several factors surrounding this process might explain the diversity of children’s reactions to divorce and the effects on them. In this study, we analyze some variables of the process of divorce from emerging adult children’s perspective and retrospective accounts, such as changes in parenting, parents’ behaviors towards the other parent in front of the child, parents’ negative emotional state and emotional instability during the divorce process, and continued conflict between parents in the post-divorce period, in order to study their association with young adult children’s attachment-related anxiety and avoidance.

### Current Study

By examining the role of parental behaviors and emotional state during the divorce process in emerging adult children’s attachment, we aimed to contribute to the literature in several ways. First, although the associations between parental divorce and adult children’s attachment have been extensively investigated, these findings have been inconclusive, mainly because, as far as we know, limited attention has been given to the study of divorce process variables, such as those referring to coparental behaviors or parents’ emotional state and instability on children’s adjustment. Furthermore, the effects of these divorce process variables have been even less widely examined on emerging adult children’s attachment representations. Finally, scarce studies have been conducted in order to investigate these effects in Spain. Thus, the main goal of this study was to analyze the association between some parental behavioral variables associated with the parental divorce process, assessed retrospectively from emerging adult children’s perspective, and young adult children’s attachment-related anxiety and avoidance. Based on the revised literature and the main goal of this study, the following hypotheses are tested:

**Hypothesis** **1** **(H1).** *Each parent’s positive behaviors during the divorce process will be associated with lower attachment-related anxiety and avoidance in young adult children. Specifically, we expected that both parents’ coparental cooperation and respect would be associated with lower attachment-related anxiety and avoidance. In addition, we expected that both parents’ continued positive parenting and freedom provided by each parent to the child to talk about the other would be associated with lower attachment-related insecurity*.

**Hypothesis** **2** **(H2).** *Both parents’ negative emotional state instability throughout the divorce process will be associated with higher attachment-related anxiety and avoidance in young adult children*.

**Hypothesis** **3** **(H3).** *Continued interparental conflict during the post-divorce period will be associated with higher attachment-related anxiety and avoidance in young adult children*.

## 2. Materials and Methods

### 2.1. Participants and Procedure

The current study is part of a larger study with a broader sample that focused on analyzing and comparing the effects of parental divorce and conflict on several social and affective variables in emerging adults from divorced and non-divorced families. This investigation only included emerging adults from divorced families. Participants with missing data or who did not meet the requirement of being young adults (i.e., between 18 and 30 years) were excluded. Hence, the analytic sample included 173 undergraduate and vocational school students from the Autonomous Community of the Basque Country. All of them belonged to a heterosexual divorced family. Mean age at time of divorce was 10.83 years. The majority of respondents reported that their mother was their main custodial parent (72.8%), followed by both parents (11.6%), the father (9.2%), and others (2.9%). The average age of respondents was 22.01 years (*SD* = 3.12), and 59.5% (*n* = 103) of participants were women. 54.4% (*n* = 94) were in a committed romantic relationship (*M* relationship duration = 34.13 months), of which 16.2% cohabited with their partner (*M* relationship duration = 50.21 months). 

Respondents were informed in class about the main goals of the study, and participated voluntarily after signing a consent form. All data were collected in person and in group. Data collection took approximately 30 min. All utilized measures were administered in Spanish. This study was approved by the Ethics Committee in Human Research at the University of the Basque Country (ethical approval code: CEISH/153/2012/SMITH ECHEBARRIA). 

### 2.2. Measures

Participants’ perceptions and recollections of parental behaviors during the divorce process were assessed through three scales, designed specifically for this study. Prior to this study, we conducted a pilot study to test the psychometric properties of these scales. After rewording some items and deleting others based on psychometric and theoretical criteria, we designed a definite measure consisting of three different scales for this study. Hereunder, we provide a description of each of these scales.

The first scale, Maternal Positive Behavioral Strategies during the Divorce Process scale (MPBSDP), consisted of nine items. An Exploratory Factor Analysis (EFA) and subsequent Confirmatory Factor Analysis (CFA) confirmed a three-factor structure. These factors include the following: maternal coparental cooperation and respect (four items; α = 0.86; e.g., “My mother conveyed respect and acceptance towards my father”); continuation of maternal positive parenting strategies (three items; α = 0.87; e.g., “My mother continued to exercise her role as a mother in the same way: helping me, loving me, paying attention to me, worrying about my problems, etc.”); and freedom provided by the mother to the child to talk about the father (two items; α = 0.90; e.g., “I could express positive feelings towards my father in front of my mother”). Fit indices for the CFA were χ^2^ (22) = 46.16; *p* < 0.01, RMSEA = 0.08; NNFI = 0.98; CFI = 0.98; and SRMR = 0.04. 

The second scale, Paternal Positive Behavioral Strategies during the Divorce Process scale (PPBSDP), consisted of nine items assessing the following three factors: paternal coparental cooperation and respect (four items; α = 0.85; e.g., “My father conveyed respect and acceptance towards my mother”); continuation of paternal positive parenting strategies (three items; α = 0.87; e.g., “My father continued to exercise his role as a father in the same way: helping me, loving me, paying attention to me, worrying about my problems, etc.”); and freedom provided by the father to the child to talk about the mother (two items; α = 0.94 e.g., “I could express positive feelings towards my mother in front of my father”). Fit indices of the CFA were χ^2^ (21) = 38.03; *p* < 0.05, RMSEA = 0.07; NNFI = 0.98; CFI = 0.99; and SRMR = 0.043. 

Finally, a third scale, named Parental Negative Emotional State (PNES), assessed both mothers’ and fathers’ negative emotional state and instability associated with divorce. A two-factor structure was confirmed through an EFA and subsequent CFA: maternal negative emotional state (four items; α = 0.90) and paternal negative emotional state (four items; α = 0.83). Sample items included “My mom/dad was sad and downcast”, “My mom/dad had frequent mood swings”. Fit indices of the CFA were χ^2^ (17) = 26.11; *p* < 0.05, RMSEA = 0.055; NNFI = 0.98; CFI = 0.99; and SRMR = 0.047. 

Post-divorce interparental conflict was assessed with a single item by asking participants to indicate the level of conflicts between their parents during the post-divorce period from 1 (= no conflicts) to 4 (= frequent conflicts).

Attachment-related anxiety and avoidance were evaluated with the Experiences in Close Relationships scale (ECR: [45]). Since our sample comprised both emerging adults involved and not involved in a committed romantic relationship, we modified some items and the instructions provided to participants, so that it could be applicable to one’s general orientation in romantic relationships, or to one’s global “attachment style” [46] (p. 84). In this sample, α for avoidance was 0.88 (18 items; e.g., “I prefer not to show a partner (significant other) how I feel deep down”) and 0.87 for anxiety (18 items; e.g., “I worry that romantic partners (others) won’t care about me as much as I care about them”). Responses to each item range from 1 (disagree strongly) to 7 (agree strongly).

Finally, some demographic characteristics, such as participants’ gender (male = 1; female = 0) and age, were accounted for as covariates. 

### 2.3. Data Analysis Plan

Statistical analyses were conducted using IBM SPSS 24, from IBM corporation (Chicago, IL, USA). Prior to the main statistical analyses, independent sample t tests were performed between those involved and not involved in a committed relationship on attachment-related avoidance and anxiety, and no significant differences were found. Comparisons were also made for sex, but no significant differences were found. Therefore, all the statistical analyses were conducted using the whole sample. First, descriptive statistics and bivariate correlations were calculated using the average score of each indicator (see Table 1 and Table 2). Next, a set of hierarchical multiple regressions were run on attachment-related anxiety and avoidance in order to analyze the predictive ability of each parent’s positive strategies towards the other during the divorce process, each parent’s negative emotional state associated with the process of divorce, and post-divorce interparental conflict. Because cross-gender parent–child dyads in family parentification processes might play a differential role in emerging adult children’s romantic relationships [47], initially, interactions with child’s gender were also tested. Given that these resulted non-significant, interactions with child’s gender were not included in our regression models. Control variables were entered in Model 1. Model 2 included the three factors associated with both maternal and paternal positive strategies during the divorce process, and the sub-scales of maternal and paternal negative emotional state related to divorce. In Model 3, post-divorce interparental conflict was added.

## 3. Results

### 3.1. Descriptive and Bivariate Analyses

Maternal positive behaviors towards the father during the divorce process to encourage the child’s positive adjustment were associated with lower attachment-related anxiety. Specifically, promoting positive relationships with the father (*r* = −0.18; *p* <0.05), the maternal positive continued role during the divorce process (*r* = −0.18; *p* < 0.05), and freedom provided to the child to talk about the father (*r* = −0.21; *p* < 0.05) were negatively correlated with lower attachment-related anxiety. 

Father’s positive behaviors towards the mother during the post-divorce process, in turn, were associated with lower attachment-related avoidance in adult children. That is, not badmouthing the mother or promoting a positive relationship with the mother (*r* = −0.20; *p* < 0.05) and the paternal positive continued role during the divorce process (*r* = −0.16; *p* <.05) were negatively correlated with lower attachment-related avoidance. 

Regarding parents’ negative emotional state and instability during the divorce process, maternal negative emotional state was associated with higher attachment-related anxiety (*r* = 0.25; *p* < 0.01). Continued interparental conflict after divorce was not significantly correlated with our outcome variables.

### 3.2. Hierarchical Multiple Regressions

#### 3.2.1. Attachment-Related Avoidance in Relationships

Results are shown in Table 3. Adult child’s gender was associated with attachment related avoidance, such that males showed greater attachment avoidance (Model 1). When both parents’ emotional state and parental behaviors associated with the process of divorce were added (Model 2), paternal coparental cooperation and respect, such as not badmouthing the mother or promoting a positive relationship with the mother, were associated with lower attachment-related avoidance (β = −0.27; *p* < 0.05). Likewise, freedom provided by the mother to their children to talk about their father was associated with lower attachment-related avoidance (β = −0.25; *p* < 0.05). These effects remained even when continued interparental conflict in the post-divorce period was added (Model 3). Contrary to our expectations, continued interparental conflict in the post-divorce period did not yield significant effects on attachment related avoidance (Model 3).

#### 3.2.2. Attachment-Related Anxiety in Relationships

Results on attachment-related anxiety are shown in Table 4. In support of our second hypothesis, when both parents’ emotional state and parental behaviors associated with the process of divorce were added, maternal negative emotional state was associated with higher attachment-related anxiety (β = 0.23; *p* < 0.05), even when continued interparental conflict in the post-divorce period was added (Model 3). In addition, freedom provided by the father to their children to talk about their mother was associated with lower attachment-related anxiety among young adult children (β = −0.24; *p* < 0.05). Again, contrary to our predictions (Hypothesis 3), continued interparental conflict in the post-divorce period was not significantly associated with higher attachment-related anxiety (Model 3).

## 4. Discussion

The main aim of this study was to examine the association between divorce process variables related to parental behaviors and young adult children’s attachment-related anxiety and avoidance. Specifically, we analyzed how coparental respect and cooperation, interparental conflict, positive parenting strategies, and both parents’ emotional state and instability in the post-divorce period are associated with children’s attachment representations during young adulthood. Even though parental divorce or separation might be positively associated with higher attachment-related anxiety and avoidance in adulthood through the development of negative schemas about intimate relationships [15], changes in parent–child relationships derived from the stress of divorce might also lead to more insecure intimate relationships in young adulthood (17). 

Prior research on the associations between parental divorce and adult children’s attachment shows inconsistent results. In fact, although some studies have found significant positive associations between parental divorce and children’s attachment, some other studies have not found such associations. For example, in a previous study [22], we found that parental divorce was not linked to higher attachment insecurity, as assessed through the attachment avoidance and anxiety dimensions. However, in the current study, which was conducted with the aim of shedding further light on these discrepancies, we did find some parental divorce process factors significantly associated with attachment-related anxiety and avoidance. Specifically, our findings indicate that both paternal and maternal strategies for children’s adequate adjustment during the divorce process influence anxious and avoidant attachment representations among adult offspring. Overall, our results suggest that both parents’ attitudes during the divorce process, whether or not they favor the adaptation of their children to this family experience, influence adult children’s level of security in their affective relationships, and particularly their level of attachment-related anxiety and avoidance in intimate relationships. Thus, from the divorce stress-adjustment perspective [25], our findings make important contributions to better comprehend the effects of parental divorce by concluding that certain family process variables might better explain emerging adult children’s reactions to divorce than divorce itself, in terms of attachment insecurity. Hereunder, we discuss in more detail these main conclusions. 

First, the findings of this study indicate that maternal negative emotional state and emotional instability during the divorce process and post-divorce period are associated with higher attachment-related anxiety among emerging adult children. That is, when children perceived that during the parental divorce process their mother was sadder, more depressed and irritable, had frequent mood swings and difficulties in controlling her emotions, young adult children reported greater attachment-related anxiety in their current intimate relationships. Maternal depression has long been related to attachment insecurity in children [48]. Moreover, maternal emotional instability may reduce her sensitivity towards her children’s signals of need, which, in turn, might cause uncertainty and anxiety in her children [48,49,50]. According to some authors, maternal sadness and instability related to divorce are associated with less availability and greater inconsistency in her responses and, as a consequence, with a lower predictability for children [43,51,52]. In this situation, children do not know to what extent they can count on their mother and, therefore, they implement hyperactivation strategies of the attachment system to call her attention, which are characteristic of the anxious attachment style [46]. Thus, the stress experienced during the divorce process can alter the behavior of parents towards their children, as well as affect their ability to respond adequately and consistently to their children’s needs for safety and protection, given that during this family process, parents are likely to be more focused on themselves than on others [8]. This finding would partially confirm our predictions regarding the association between both parents’ emotional instability and emerging adult children’s attachment-related anxiety and avoidance (Hypothesis 2), since such results were not significant for attachment avoidance nor regarding the father’s negative emotional state or emotional instability. This might be due to the fact that mothers are usually the main attachment figures and they frequently hold their children’s primary custody. Furthermore, divorced mothers who mainly parent on their own tend to experience higher levels of stress because they cannot count on a partner for financial or social support. This heightened strain might lead to increased levels of depressive symptoms, which may negatively affect the quality of mother–child interactions [53]. Hence, it is more likely that mothers’ behaviors towards their children due to their emotional instability in the post-divorce period might be more influential than that of fathers on their children’s attachment. In fact, bivariate correlations revealed that higher maternal positive parenting in the post-divorce period led to lower attachment-related anxiety. In addition, in agreement with our first hypothesis, paternal positive attitudes during the divorce process, such as providing the child with the freedom to talk about his or her mother, were associated with lower attachment-related anxiety in emerging young adult children. 

Regarding attachment-related avoidance, our results suggest that when the father facilitated a good relationship between the child and the mother, transmitting respect and acceptance towards her and not damaging her image, adult children showed lower attachment avoidance in their affective relationships. Additionally, the freedom provided to the child by the mother to talk about the father was associated with lower attachment-related avoidance. These results again confirm our first hypothesis related to the association between each parent’s positive behaviors during the divorce process and the post-divorce period and young adult children’s attachment-related avoidance. 

The results of this study are consistent with previous studies which suggest that in divorced families where parents try to turn the child against the other parent by making negative comments about him or her, entrusting the child with issues related to the divorce, or asking the child to be in favor of one parent or the other, by encouraging the child to ignore or disrespect the rules and authority of the other parent, children both in the short and the long term show lower attachment security [54,55,56,57]. As a result, these negative parental strategies may lead their children to think that the other parent is insecure, unavailable, or unloving, and that he or she rejects him or her, which could generate more negative mental representations about intimate relationships. 

Finally, contrary to hypotheses, no associations were found between high interparental conflict in the post-divorce period and children’s attachment-related anxiety and avoidance. The lack of significant findings regarding the influence of high interparental conflict might be because our measure of post-divorce interparental conflict did not assess prominent dimensions of interparental conflict (i.e., frequency, intensity, resolution) that are associated with children’s maladjustment [58]. 

### Study Limitations, Strengths, and Implications

Our results should be seen in light of several limitations. First, this study had a cross-sectional design. This makes it difficult to infer a direct temporary link between past parental strategies during divorce and the post-divorce period and their offspring’s current attachment-related security or insecurity level. That is, we cannot confirm that these parental post-divorce strategies are the cause of adult children’s attachment-related anxiety or avoidance. Therefore, our results should be interpreted with caution and verified with a longitudinal examination of these variables. Second, because we only analyzed college and vocational school students, this study may prevent generalization to other populations. 

Third, our measure of post-divorce interparental conflict consisted of a single Likert-type item in which young adults were asked to indicate the level of conflict between their parents. Parental conflict is a complex phenomenon which has been defined from different theoretical approaches and through multiple dimensions, such as frequency, intensity, or resolution of conflicts between parents. Therefore, future studies should focus on analyzing the effects of these dimensions of interparental conflict in post-divorced families. In addition, even though different family types and structures were not excluded in this investigation, this study only included heterosexual families. Thus, this investigation should be replicated with different family structures, in order to verify differences and similarities among these types of families. 

Likewise, the majority of the analyzed children (72.8%) had their mother as the main custodial parent. Hence, further research is needed with more children of divorce being raised in shared custody and with their fathers, to control for the effects of different custody types. 

Next, prior research demonstrates that heightened stress related to divorce predicts lower emotional well-being and higher levels of depression among parents [53]. In the current study, we did not examine specific stressors surrounding divorce that may precipitate changes in parental behaviors or emotional well-being. Future studies should therefore replicate this study by examining stressors surrounding the divorce experience that may cause variations in parental roles, in order to determine whether it mediates children’s adjustment. 

Moreover, our study only focused on adult children’s retrospective post-divorce accounts. Thus, for a clearer picture of post-divorced families, it would also be useful to gather information from the parents’ perspective. Finally, although the time elapsed since divorce has frequently been analyzed as a control variable in several studies focused on analyzing the effects of divorce on children, it was not examined in our research work. Therefore, this limitation should be overcome by replicating this study with also controlling for time since divorce.

Despite these limitations, our study contributes to the literature in several ways. First, from the divorce stress-adjustment perspective, it focuses on the examination of the influence of family life changes and stressful circumstances surrounding the divorce process and the post-divorce period on emerging adults’ current attachment-related anxiety and avoidance. In particular, we examined parental attitudes that facilitate or hinder children’s adjustment to this family experience. These include each parent’s coparental respect and cooperation, the freedom provided by each parent to the child in order to express positive feelings and to talk about the other parent, the maintenance of unconditional support and positive parenting with respect to their children, and each parent’s emotional state and stability during the separation process and post-divorce period. Next, this investigation analyzes a non-widely studied developmental stage and population of emerging adults from a country where divorce is a relatively recent social and family event. Finally, another noteworthy contribution refers to the study of the independent effects of mothers’ and fathers’ post-divorce behaviors, as most studies have focused on analyzing both parents’ coparenting behaviors as a whole. 

The findings of this investigation also have several implications for public policies and preventive interventions for divorced families with dysfunctional family dynamics. Our study demonstrates that positive relationships between separated or divorced parents, such as maintaining a coparental relationship based on mutual respect and cooperation, and both parents’ post-divorce emotional stability play a decisive role in emerging adults’ affective relationships. Indeed, a number of studies show that children’s well-being in the post-divorce period has less to do with family structure in itself, but rather with increased family strain and changes in coparental relationships [53,59]. In this vein, it is of vital importance to support and provide tools for parents in order to maintain a coparental relationship based on cooperation and respect in the post-divorce period. The relevance of coparental cooperation is supported and emphasized by the “Shared Custody” law, approved in the Basque Autonomous Community in 2011, in which the child’s well-being and interests are defended above all by highlighting parental co-responsibility and the right of children to be raised by and relate to each parent [60]. Thus, given that our participants belong to this community and based on our findings, more prevention and intervention programs should be designed and implemented aimed at training parents to maintain a positive coparental relationship and emotional stability in order to prevent children’s exposure to possible loyalty conflicts after parental separation or divorce, especially in this cultural context. 

## 5. Conclusions

The data obtained in our study show that maternal emotional state and stability, positive communication about the other parent, and a greater capacity to control anger and maintain a positive exchange of information with respect to the other parent predict secure affective relationships in emerging adult children [39]. These types of positive behaviors promoted by parents allow children to feel loved and cared for by both parents, which set an essential foundation in the development of secure attachment relationships [61].

## Figures and Tables

**Table 1:** Descriptive information for study variables.

Constructs	Indicators	*M (SD)*	Range	α
Maternal positive behaviors	Maternal coparental coop.	4.39 (1.44)	1–6	0.86
	Maternal positive parenting	4.69 (1.39)	1–6	0.87
	Freedom mother father	4.46 (1.59)	1–6	0.90
Paternal positive behaviors	Paternal coparental coop.	4.36 (1.44)	1–6	0.85
	Paternal positive parenting	3.80 (1.68)	1–6	0.87
	Freedom father mother	4.11 (1.70)	1–6	0.94
Parental negative emotional state	Mother	3.36 (1.56)	1–6	0.90
	Father	3.00 (1.40)	1–6	0.83
Continued post-divorce interparental conflict	--	2.39 (1.16)	1–4	-
Current attachment	Avoidance	2.51 (0.94)	1–7	0.88
	Anxiety	3.62 (0.99)	1–7	0.87

Maternal coparental coop. = maternal coparental cooperation and respect; Maternal positive parenting = continuation of maternal positive parenting strategies; Freedom mother father = freedom provided by the mother to talk about the father; Paternal coparental coop. = paternal coparental cooperation and respect; Paternal positive parenting = continuation of paternal positive parenting strategies; Freedom father mother = Freedom provided by the father to talk about the mother.

**Table 2:** Correlations among the study variables.

	1	2	3	4	5	6	7	8	9	10	11
1. Maternal coparental coop	1										
2. Maternal positive parenting	0.59 **	1									
3. Freedom mother father	0.66 **	0.59 **	1								
4. Paternal coparental coop.	0.36 **	0.21 **	0.17 *	1							
5. Paternal positive parenting	0.04	0.09	0.25 **	0.50 **	1						
6. Freedom father mother	0.17 *	0.27 **	0.46 **	0.52 **	0.66 **	1					
7. Maternal negative emo. state	−0.50 **	−0.62 **	−0.37 **	−0.18 *	−0.10	−0.14	1				
8. Paternal negative emo. state	−0.12	−0.07	0.01	−0.55 **	−0.45 **	−0.40 **	0.22 **	1			
9. Post-divorce interparental conf.	−0.32 **	−0.08	−0.16 *	−0.39 **	−0.29 **	−0.28 **	0.20 *	0.25 **	1		
10. Attachment avoidance	−0.04	−0.09	−0.13	−0.20 *	−0.16 *	−0.15	0.09	0.11	0.02	1	
11. Attachment anxiety	−0.18 *	−0.18 *	−0.21 **	−0.07	0.001	−0.14	0.25 **	0.07	0.07	0.33 **	1

Maternal coparental coop. = maternal coparental cooperation and respect; Maternal positive parenting = continuation of maternal positive parenting strategies; Freedom mother father = freedom provided by the mother to talk about the father; Paternal coparental coop. = paternal coparental cooperation and respect; Paternal positive parenting = continuation of paternal positive parenting strategies; Freedom father mother = freedom provided by the father to talk about the mother; Maternal negative emo. state = maternal negative emotional state; Paternal negative emo. state = paternal negative emotional state; Post-divorce interparental conf. = post-divorce interparental conflict. * *p* < 0.05, ** *p* < 0.01.

**Table 3:** Summary of hierarchical multiple regression for some variables related to the parental divorce process predicting attachment-related avoidance.

	Model 1			Model 2			Model 3		
Variable	*B*	*SE B*	β	*B*	*SE B*	β	*B*	*SE B*	β
Control variables									
Gender (male)	0.44	0.15	0.23 **	0.51	0.15	0.27 ***	0.50	0.15	0.26 ***
Age	0.04	0.03	0.13	0.03	0.02	0.08	0.03	0.02	0.09
Explanatory variables									
Maternal coparental cooperation				0.17	0.08	0.27	0.15	0.09	0.24
Maternal positive parenting strategies				−0.003	0.08	−0.005	0.01	0.08	0.02
Freedom mother father				−0.15	0.08	−0.26 *	−0.15	0.08	−0.25 *
Paternal coparental cooperation				−0.17	0.08	−0.26 *	−0.17	0.08	−0.27 *
Paternal positive parenting strategies				−0.07	0.06	−0.13	−0.08	0.06	−0.14
Freedom father mother				0.05	0.07	0.10	0.05	0.07	0.08
Mother’s negative emotional state				0.04	0.06	0.07	0.05	0.06	0.08
Father’s negative emotional state				−0.04	0.07	−0.06	−0.04	0.07	−0.06
Post-divorce interparental conflict							−0.06	0.07	−0.07
*R* ^2^	0.07			0.16			0.16		
*F* for change in *R*^2^	3.49 *			2.33 **			2.18 *		

* *p* < 0.05, ** *p* < 0.01, *** *p* < 0.001.

**Table 4:** Summary of hierarchical multiple regression for some variables related to the parental divorce process predicting attachment-related anxiety.

	Model 1			Model 2			Model 3		
Variable	*B*	*SE B*	β	*B*	*SE B*	β	*B*	*SE B*	β
Control variables									
Sex (male)	−0.14	0.16	−0.07	−0.17	0.16	−0.09	−0.18	0.16	−0.09
Age	0.04	0.03	0.12	0.02	0.03	0.08	0.03	0.03	0.08
Explanatory variables									
Maternal coparental cooperation				−0.01	0.09	−0.01	−0.03	0.09	−0.04
Maternal positive parenting strategies				0.07	0.09	0.10	0.08	0.09	0.12
Freedom mother father				−0.09	0.08	−0.14	−0.08	0.08	−0.13
Paternal coparental cooperation				0.004	0.08	0.006	−0.001	0.08	−0.001
Paternal positive parenting strategies				0.10	0.07	0.18	0.09	0.07	0.17
Freedom father mother				−0.13	0.07	−0.22 *	−0.13	0.07	−0.24 *
Mother’s negative emotional state				0.13	0.07	0.22 *	0.14	0.07	0.23 *
Father’s negative emotional state				−0.01	0.07	−0.02	−0.01	0.07	−0.02
Post-divorce interparental conflict							−0.06	0.08	−0.07
*R* ^2^	0.02			0.12			0.13		
*F* for change in *R*^2^	1.19			1.71 *			1.61		

* *p* < 0.05

## Data Availability

The data presented in this study are available on request from the corresponding author.
